# Low-level endemic circulation of porcine reproductive and respiratory syndrome virus in slaughtered pigs from Meghalaya, Northeast India: A cross-sectional seroepidemiological study

**DOI:** 10.14202/vetworld.2026.1043-1051

**Published:** 2026-03-15

**Authors:** Arockiasamy Arun Prince Milton, Kandhan Srinivas, Sabia Khan, Sharanagouda S Patil, Kekungu Puro, Sandeep Ghatak, Vivek Joshi, Samir Das

**Affiliations:** 1 Indian Council of Agricultural Research – Research Complex for North Eastern Hill Region, Umiam, Meghalaya, India; 2 Indian Council of Agricultural Research–National Institute of Veterinary Epidemiology and Disease Informatics, Bengaluru, Karnataka, India

**Keywords:** backyard pig farming, enzyme-linked immunosorbent assay, Meghalaya, Northeast India, porcine reproductive and respiratory syndrome virus, seroepidemiology, slaughterhouse surveillance, swine health

## Abstract

**Background and Aim:**

Porcine reproductive and respiratory syndrome (PRRS) is a major transboundary viral disease of pigs, causing substantial economic losses worldwide. Repeated outbreaks and molecular evidence from Northeastern states indicate endemic virus circulation. Meghalaya, where pig production is dominated by smallholder and backyard systems with limited biosecurity, remains poorly characterized from a seroepidemiological perspective. This study aimed to estimate the apparent and true seroprevalence of PRRS virus in slaughtered pigs from Meghalaya and to evaluate selected animal-level risk factors.

**Materials and Methods:**

A cross-sectional serosurvey was conducted between August 2023 and July 2024 at an organized slaughterhouse in Shillong, Meghalaya. A total of 413 serum samples were collected from apparently healthy pigs originating from four districts: Ri Bhoi, East Khasi Hills, Eastern West Khasi Hills, and West Jaintia Hills. Sera were screened for virus-specific immunoglobulin G antibodies using the HerdChek® PRRS X3 Antibody Test based on the enzyme-linked immunosorbent assay. Apparent seroprevalence and corresponding 95% confidence intervals (CIs) were calculated using the Agresti–Coull method, while true prevalence was estimated using the Rogan–Gladen estimator. Univariate analysis was performed to assess associations between seropositivity and age, sex, breed, and district of origin using chi-square or Fisher’s exact tests, with odds ratios and 95% CIs used to quantify associations.

**Results:**

Out of 413 serum samples tested, 21 were seropositive, resulting in an apparent seroprevalence of 5.08% (95% CI: 3.31–7.69). The estimated true prevalence was 3.94% (95% CI: 2.98–6.60). District-wise seroprevalence ranged from 1.23% to 8.85%, with the highest proportion of seropositive animals observed in East Khasi Hills. No statistically significant associations were detected between seropositivity and age, sex, or breed (*p* > 0.05).

**Conclusion:**

The detection of antibodies in slaughtered pigs confirms low-level endemic circulation of the virus in Meghalaya. Although the observed prevalence was moderate, the findings indicate a latent risk of future outbreaks under prevailing low biosecurity production systems. Sustained surveillance, strengthened biosecurity measures, and integration of serological monitoring into regional disease control programs are essential to safeguard pig health, rural livelihoods, and food security in Northeast India.

## INTRODUCTION

Northeast India accounts for approximately 46.85% of India’s total pig population, with Meghalaya contributing 7.79% [1]. The Northeastern region (NER) shares porous international borders with five countries [2]. The region is predominantly inhabited by tribal communities, many of whom rear pigs under low-input production systems that rely on locally available resources [3]. Pig farming represents a primary source of livelihood and holds considerable sociocultural significance for these indigenous populations [3]. However, the piggery sector in this region remains underdeveloped, particularly with respect to disease control measures, including biosecurity, modern disease management, and waste disposal systems. This structural vulnerability is reflected in the recent emergence and spread of major transboundary viral diseases (TBVDs), notably porcine reproductive and respiratory syndrome (PRRS) and African swine fever (ASF) [4].

PRRS is a highly contagious TBVD that severely affects the pig industry and results in substantial economic losses. The disease is caused by an RNA virus belonging to the family Arteriviridae and the genus Betaarterivirus, and is characterized by reproductive failure in sows and severe respiratory illness with high mortality in younger pigs [5, 6]. Transmission occurs through direct contact with infected animals and indirectly via aerosols, contaminated transport vehicles, semen, insects, and fomites [7]. India was considered free from PRRS until the first reported outbreak in Mizoram, a Northeastern state, on June 26, 2013 [8]. Following the initial outbreak of highly pathogenic PRRS, the disease has continued to re-emerge in sporadic outbreaks and has now assumed endemic status in the region [6–8]. Molecular evidence indicates that PRRS virus isolates circulating in India are closely related to strains reported from neighboring countries such as China, Myanmar, Bhutan, and Nepal [6].

ASF has recently entered India through the NER, resulting in high mortality and significant trade disruptions. Notably, a mixed infection involving PRRS virus (PRRSV) genotype 2 and ASF virus (ASFV) genotype II was reported in Assam, a state bordering Meghalaya, highlighting the severity of the epidemiological situation and the heightened risk of TBVD mortality [6]. The PRRSV genotype 2 (North American type) circulating in India is associated with more severe respiratory disease compared with genotype 1 (European type) [7]. Although a comprehensive economic loss analysis for PRRS has not yet been conducted in India, data from China indicate an estimated economic impact of ¥1,424.37 per sow. In breeding herds, losses are primarily attributed to reduced numbers of weaned piglets, whereas in fattening herds, increased feed costs account for the largest proportion of losses (44.88%) [9]. Given that China is heavily affected by PRRS and shares an extensive border with India, these figures underscore the potential economic impact of the disease within the Indian pig production system.

Despite repeated outbreaks and increasing molecular evidence of PRRS circulation in the NER, systematic seroepidemiological data from Meghalaya remain scarce. Most available studies from India have focused on outbreak investigations or molecular characterization of PRRSV, with limited emphasis on population-level seroprevalence, particularly in states dominated by backyard and smallholder pig production systems. Furthermore, existing serosurveys from the NER are either outdated, geographically limited, or restricted to organized farms, which do not adequately represent the epidemiological situation in regions where informal pig trade, low biosecurity, and close animal–human–environment interactions prevail. The recent incursion of ASF into the NER and reports of PRRSV–ASFV co-infections further underscore the need to understand the baseline exposure of pig populations to PRRSV, as such TBVDs may act synergistically to exacerbate disease impact. In Meghalaya, data on district-wise seroprevalence and animal-level risk factors derived from representative slaughterhouse sampling are notably lacking, creating a critical knowledge gap that limits evidence-based surveillance and control strategies.

The present study was therefore designed to estimate the apparent and true seroprevalence of PRRSV among slaughtered pigs originating from major pig-rearing districts of Meghalaya in the NER. In addition, the study aimed to evaluate selected animal-level factors, including age, sex, breed, and district of origin, for their association with PRRSV seropositivity. By generating updated seroepidemiological evidence from a slaughterhouse-based cross-sectional survey, this study seeks to provide baseline data to inform PRRS surveillance, support early warning systems, and guide integrated control measures for PRRS and other TBVDs such as ASF in smallholder-dominated pig production systems.

## MATERIALS AND METHODS

### Ethical approval

The study protocol was reviewed and approved by the Institute Animal Ethics Committee (IAEC) of the Indian Council of Agricultural Research – Research Complex for North Eastern Hill Region (ICAR–RC NEH), Umiam, Meghalaya, India.

The IAEC is registered with the Committee for Control and Supervision of Experiments on Animals (CCSEA), Ministry of Fisheries, Animal Husbandry and Dairying, Government of India, under approval reference number V-1101(13)/122023-CPCSEA-DADF dated December 5, 2023.

The study was conducted in accordance with the guidelines of CCSEA for the care and use of animals in scientific research and complied with national regulations governing animal experimentation and biosafety in India. As sampling was performed at a licensed and organized slaughterhouse, no live experimental infection, invasive surgical procedures, or experimental manipulation of animals were carried out.

Blood samples were collected from apparently healthy pigs during routine slaughter operations. Sampling was performed immediately post-exsanguination to avoid additional handling stress or procedural discomfort. No animal was subjected to procedures beyond standard slaughterhouse practice for the purpose of this study. Therefore, the study involved minimal ethical risk and did not alter normal animal management or slaughter procedures.

Prior permission was obtained from slaughterhouse authorities before sample collection. Verbal consent was also obtained from slaughterhouse personnel responsible for animal handling and documentation. Only pigs originating from districts within Meghalaya were included in the study to maintain epidemiological consistency and traceability.

All laboratory procedures involving serum handling and serological testing were conducted in accordance with the institutional biosafety protocols of ICAR RC NEH. Samples were transported and stored under appropriate cold-chain conditions to ensure integrity and biosecurity compliance. The study did not involve zoonotic risk exposure to personnel, and standard personal protective equipment and biosafety practices were followed throughout sample collection and laboratory analysis.

As PRRS is not a zoonotic disease, the study did not pose direct public health risks. However, ethical consideration was given to the broader implications for animal health, rural livelihoods, and food security in Northeast India.

### Study period and location

**Figure 1:**
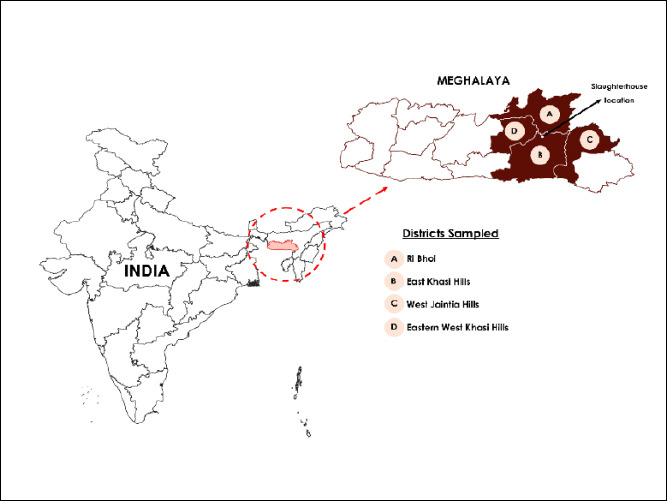
Map showing the districts of Meghalaya from which pigs were sampled [Source: https:// github.com/ datta07/INDIAN-SHAPEFILES].

This cross-sectional serosurvey was conducted between August 2023 and July 2024. Samples were collected from an organized slaughterhouse located in Shillong, Meghalaya (Latitude 25.60815° N, Longitude 91.87409° E). The slaughterhouse receives pigs from multiple districts across Meghalaya, primarily East Khasi Hills, West Khasi Hills, Ri Bhoi, and West Garo Hills (Figure 1), as well as from neighboring states. These districts represent diverse agro-climatic zones ranging from subtropical high-rainfall plateaus to humid low-lying foothills. Pig population density is highest in Ri Bhoi and West Garo Hills, where backyard and smallholder pig farming constitute major livelihood activities. Traditional pig-rearing systems predominate and are characterized by low input, minimal veterinary intervention, and reliance on locally available feed resources. The Khasi, Jaintia, and Garo tribes are the principal pig-rearing communities in Meghalaya.

### Serum sample collection

According to the 20th Livestock Census of India, the total pig population in Meghalaya is 706,364. The required sample size for estimating PRRS seroprevalence was calculated as 213 using a standard prevalence-based formula [10, 11]. The sample size (n) was calculated as follows:

N·Z²·P·(1−P)/[d²·(N−1)+Z²·P·(1−P)],

where N is the population size (706,364), P is the expected prevalence (3.66%), d is the desired precision (±2.5%), and Z is the standard normal deviation at 95% confidence (1.96) [12]. Although the estimated minimum sample size was 213, a total of 413 samples were collected to increase statistical power and improve precision.

**Figure 2:**
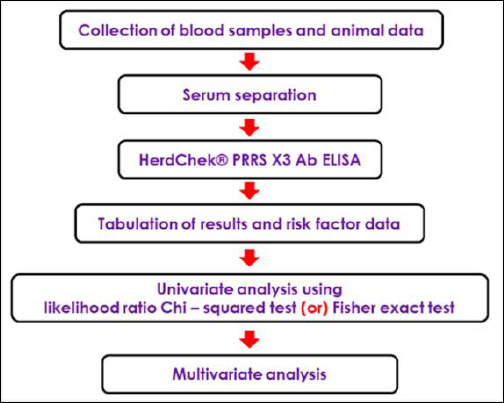
Flow chart indicating the collection of samples and subsequent data analysis.

Blood samples were collected aseptically from apparently healthy pigs during slaughter. Only pigs originating from four districts of Meghalaya, namely Ri Bhoi, East Khasi Hills, Eastern West Khasi Hills, and West Jaintia Hills, were included. The district of origin was confirmed through slaughterhouse personnel, and given that only 5–10 animals are slaughtered daily, traceability was considered reliable. Blood was collected into sterile serum vacutainers and transported to the laboratory under refrigerated conditions. Sera were separated by centrifugation at 500 × *g* for 15 min, transferred to microfuge tubes, and stored at −20°C until testing. All samples were analyzed within 2 weeks of collection. As PRRS vaccination is not practiced in India, none of the sampled animals had been vaccinated. Information on management-related risk factors, including housing, herd size, and farm-level biosecurity, could not be obtained because sampling was performed at slaughterhouses. A schematic flow chart of sample collection and data analysis is shown in Figure 2.

### Serological analysis

Serum samples were tested for PRRSV-specific antibodies using the HerdChek® PRRS X3 Ab enzyme-linked immunosorbent assay kit (IDEXX Laboratories, Inc., Westbrook, ME, USA; catalog no. 99-18070) according to the manufacturer’s instructions. A sample-to-positive (S/P) ratio cutoff of 0.4 was used to classify samples as positive or negative. The reported diagnostic sensitivity and specificity of the kit are 98.8% and 99.9%, respectively [13]. Positive and negative controls supplied by the manufacturer were included on each plate; duplicate testing was not performed as it was not recommended by IDEXX Laboratories.

### Statistical analysis

Attribute-wise seroprevalence and corresponding 95% confidence intervals (CI) were calculated using the Agresti–Coull method [14]. True prevalence was estimated using the Rogan–Gladen estimator [15], and 95% confidence limits were derived using the Wilson method [16]. These calculations were performed using an online epidemiological tool (https://epitools.ausvet.com.au/trueprevalence), accessed on March 19, 2025. The dataset was checked for completeness, and no missing values were identified.

Univariate associations between PRRSV seropositivity and explanatory variables were assessed using the likelihood ratio chi-square test, while Fisher’s exact test was applied when chi-square assumptions were not met. A p-value < 0.05 was considered statistically significant. The strength of associations was expressed as odds ratios with 95% CIs. Variables with a p-value < 0.25 in univariate analysis were considered for inclusion in multivariate analysis. Multivariate modeling was performed using a forward stepwise likelihood ratio approach, guided by the lowest Akaike information criterion value. Model fit was assessed using the likelihood ratio chi-square goodness-of-fit test. All analyses were conducted using RStudio v 2025.05.0 [17], build 496, running R version 4.4.1 [18], with the packages epiDisplay v3.5.0.2 [19] and vcd v1.4-13 [20].

## RESULTS

### Apparent and true PRRS seroprevalence

The seroprevalence of PRRS infection in slaughtered pig samples from Meghalaya, Northeastern India, was determined using a commercial enzyme-linked immunosorbent assay kit. Of the 413 serum samples tested, 21 animals were seropositive, corresponding to an apparent seroprevalence of 5.08%. The apparent and true prevalence estimates were 5.08% (95% CI: 3.31–7.69) and 3.94% (95% CI: 2.98–6.60), respectively. No equivocal results were recorded, as samples with a sample-to-positive ratio ≥0.4 were classified as positive, whereas those with values <0.4 were considered negative in accordance with the manufacturer’s criteria.

### Risk factor analysis

The demographic characteristics of the sampled pigs are summarized in Table 1. The serum samples comprised 301 males (72.88%) and 112 females (27.12%). Based on age, 53 animals (12.83%) were <6 months old, 308 animals (74.58%) were 7–12 months old, and 52 animals (12.59%) were >12 months old. The mean age of the sampled population was 9.27 months, with individual ages ranging from 5 months to >24 months. For analytical clarity, pigs were categorized into three age groups: 0–6 months, 7–12 months, and >12 months.

**Table 1:** Demographic attributes of the screened pigs.

Attribute	Number of animals	Percentage (pigs/total)
Overall	413	100.00
Age		
0–6 months	53	12.83 (53/413)
7–12 months	308	74.58 (308/413)
Above 12 months	52	12.59 (52/413)
Sex		
Male	301	72.88 (301/413)
Female	112	27.12 (112/413)
Origin		
Ri Bhoi	92	22.28 (92/413)
East Khasi Hills	113	27.36 (113/413)
West Jaintia Hills	81	19.61 (81/413)
Eastern West Khasi Hills	127	30.75 (127/413)
Breed		
Local	200	48.43 (200/413)
Crossbred	213	51.57 (213/413)

**Figure 3:**
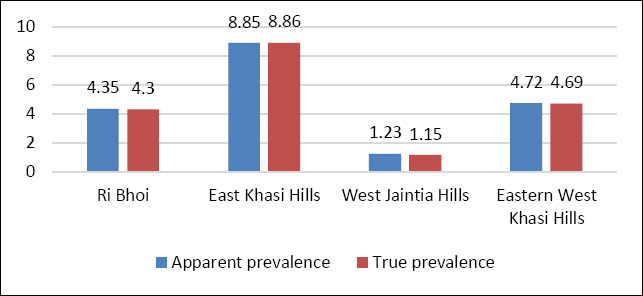
Bar chart depicting the district-wise seroprevalence rates of the samples.

With respect to breed, 200 animals (48.43%) were local pigs, while 213 animals (51.57%) were crossbred. The overall apparent seroprevalence of PRRS was 5.08% (95% CI: 3.31–7.69), with a corresponding true prevalence of 3.94% (95% CI: 2.98–6.60). District-wise PRRSV seroprevalence varied, with values of 4.35% in Ri Bhoi (4/92), 8.85% in East Khasi Hills (10/113), 1.23% in West Jaintia Hills (1/81), and 4.72% in Eastern West Khasi Hills (6/127) (Table 2; Figure 3).

**Table 2:** Univariate analysis of porcine reproductive and respiratory syndrome virus seroprevalence.

Attribute	Number of animals	Percentage positive (n/N)	95% CI (2.5%)	95% CI (97.5%)	Odds ratio	95% CI (2.5%)	95% CI (97.5%)	p-value
Age								0.530^a^
0–6 months	53	1.89 (1/53)	0.00	10.88	0.310	0.017	1.551	0.259
7–12 months	308	5.84 (18/308)	3.67	9.10	0.644	0.101	2.327	0.564
Above 12 months	52	3.85 (2/52)	0.32	13.72	Reference	Reference	Reference	Reference
Sex								0.723^b^
Male	301	5.32 (16/301)	3.24	8.52	1.201	0.458	3.745	0.727
Female	112	4.46 (5/112)	1.66	10.29	Reference	Reference	Reference	Reference
Origin								0.119^a^
Ri Bhoi	92	4.35 (4/92)	1.36	11.00	0.469	0.125	1.453	0.213
East Khasi Hills	113	8.85 (10/113)	4.71	15.69	Reference	Reference	Reference	Reference
West Jaintia Hills	81	1.23 (1/81)	0.00	7.32	0.129	0.007	0.693	0.053
Eastern West Khasi Hills	127	4.72 (6/127)	1.97	10.14	0.511	0.169	1.423	0.208
Breed								0.328^b^
Local	200	4.00 (8/200)	1.91	7.82	0.641	0.249	1.555	0.334
Crossbred	213	6.10 (13/213)	3.51	10.25	Reference	Reference	Reference	Reference

a p-values calculated using Fisher’s exact test (assumptions for chi-squared test violated).

b p-values calculated using the likelihood ratio chi-square test. CI = Confidence interval.

Univariate analysis revealed variation in odds ratios across age, sex, breed, and district of origin; however, none of these variables showed a statistically significant association with PRRS seropositivity (*p* > 0.05) (Table 2). All variables were initially considered for multivariate model fitting, but no model with adequate statistical support based on Akaike information criterion and likelihood ratio values could be obtained due to the absence of significant associations at the univariate level. Consequently, the results of the multivariate analysis are not presented, as they did not yield any meaningful or statistically significant findings.

## DISCUSSION

### Epidemiological interpretation of PRRS seroprevalence

Owing to the high genetic variability and frequent recombination events observed among PRRSV strains, no universally effective vaccine is currently available for disease prevention [21]. When combined with suboptimal biosecurity practices commonly associated with small-scale and backyard pig holdings, PRRS represents a substantial threat to the piggery sector, which is largely sustained by economically vulnerable communities in India. The present study provides an updated seroepidemiological assessment of PRRS in slaughtered pigs from Meghalaya, Northeast India. An overall apparent seroprevalence of 5.08% and a corresponding true prevalence of 3.94% were recorded, indicating ongoing virus circulation. Among the surveyed districts, East Khasi Hills exhibited the highest seroprevalence (8.85%). This observed heterogeneity is more likely attributable to variations at the household or farm-level rather than true district-level differences, as sampling was limited to apparently healthy animals presented for slaughter. Notably, the current seroprevalence exceeds earlier reports from Meghalaya, which documented prevalence rates of 2.8% in 2014, 2.7% in 2015, and 3.62% in 2016 [12].

### Comparison with regional and national studies

Comparable serological investigations from other Indian states have reported variable prevalence estimates. In Assam, a seroprevalence of 0.84% (9/1064) was documented among sows and gilts with reproductive disorders between 2013 and 2016 [22]. In contrast, a markedly higher seroprevalence of 27.86% (117/420) was reported from Mizoram during 2018–19 [23], while organized swine farms in Punjab showed a seroprevalence of 23.3% (21/90) [24]. In addition, molecular and pathological evidence of PRRS has been reported from Kerala, Assam, and Mizoram, confirming virus circulation across diverse pig-rearing systems in India [6, 7, 25–29]. Of particular concern, a natural outbreak of highly pathogenic PRRS in wild pigs in Mizoram highlights the potential for wildlife spillover and the associated threat to wild swine populations [30].

### Diagnostic considerations and serological approach

The non-specific clinical presentation and frequent subclinical course of PRRS necessitate laboratory-based diagnostics for accurate detection and surveillance [30]. Indirect enzyme-linked immunosorbent assay remains the most widely used method for population-level antibody detection [30]. The IDEXX kit employed in the present study is recognized for its high sensitivity and specificity and is considered a reference assay for PRRS serosurveillance [13, 21, 30, 31]. Most commercial assays, including the IDEXX kit, target the PRRSV nucleocapsid protein encoded by the ORF7 gene. This protein is relatively conserved, constitutes approximately 20%–40% of the viral particle, and exhibits strong antigenicity and immunogenicity, making it suitable for serological detection [30].

### Risk factor interpretation, limitations, and One Health relevance

No statistically significant associations were observed between PRRS seroprevalence and age, sex, or breed, suggesting relatively uniform exposure across pig populations. This pattern may reflect widespread virus circulation or shared risk factors such as pig trade movements and inadequate farm-level biosecurity. However, the use of slaughterhouse-based sampling, which aggregates animals from multiple sources, may mask finer epidemiological patterns. Although Meghalaya relies heavily on pig importation to meet local demand, only pigs sourced within the state were included in this study. Given confirmed PRRS activity in neighboring Northeastern states, future studies incorporating imported pigs are warranted to better elucidate regional transmission dynamics.

Several limitations should be acknowledged. Sampling was restricted to slaughtered pigs and may not fully represent live herd-level epidemiology. Seropositive samples were not confirmed by molecular assays, and risk factor analysis was limited to animal-level variables due to the unavailability of farm management data. Moreover, the influence of informal pig trade networks and cross-border movements was not assessed. Although PRRS is not zoonotic, its interaction with ASF poses serious socio-economic risks to tribal communities dependent on pig farming, underscoring the broader One Health implications linking animal health, livelihoods, and food security.

## CONCLUSION

This study provides updated seroepidemiological evidence of PRRS circulation in slaughtered pigs from Meghalaya, NER. An apparent seroprevalence of 5.08% and a true prevalence of 3.94% were recorded, confirming low-level but persistent PRRSV circulation in the region. District-wise variation was observed, with East Khasi Hills showing the highest seroprevalence (8.85%). No statistically significant associations were detected between PRRS seropositivity and age, sex, or breed, suggesting relatively uniform exposure across pig populations.

The detection of PRRSV antibodies in apparently healthy slaughtered pigs highlights the silent circulation of the virus under predominantly low input and low biosecurity production systems. These findings underscore the need for sustained serological surveillance integrated into routine animal health monitoring programs. Strengthening biosecurity at farm-level, improving traceability of pigs entering slaughterhouses, and enhancing surveillance at animal movement and entry points are critical to reducing PRRSV spread. Given the concurrent circulation of ASF in the NER, coordinated control strategies targeting multiple TBVDs are essential to safeguard pig health and rural livelihoods.

Key strengths include the use of a robust sample size exceeding the calculated minimum requirement, slaughterhouse-based sampling that captures pigs from multiple districts, and estimation of both apparent and true prevalence using validated statistical methods. The application of a highly sensitive and specific serological assay further enhances the reliability of the prevalence estimates.

The study was limited to slaughterhouse samples, which may not fully represent herd-level infection dynamics in live farms. Imported pigs were excluded, despite their importance in meeting local pork demand. Seropositive samples were not confirmed by molecular assays, and risk factor analysis was restricted to animal-level variables because farm management and biosecurity data were unavailable. In addition, informal pig trade networks and cross-border movements were not assessed.

Future investigations should focus on longitudinal, farm-based serosurveys combined with molecular detection and sequencing of PRRSV to elucidate transmission pathways and viral evolution. Inclusion of imported pigs and assessment of pig movement networks would provide a more comprehensive understanding of PRRS epidemiology in the NER. Evaluating co-infections with ASF and other endemic pathogens will also be critical for designing integrated disease control strategies within a One Health framework.

Overall, the study confirms ongoing PRRSV circulation in Meghalaya and highlights a latent risk of larger outbreaks under prevailing production systems. Sustained surveillance, improved biosecurity, and evidence-based control measures are imperative to mitigate the impact of PRRS and associated TBVDs on pig production, food security, and the socio-economic well-being of tribal communities in the NER.

## DATA AVAILABILITY

All the generated data are included in the manuscript.

### AUTHORS’ CONTRIBUTIONS

AAPM and SD: Conceptualization, methodology, and drafted the manuscript. AAPM, KS, and SK: Laboratory investigation. KS, AAPM, SD, SSP, SG, KP, and VJ: Analysis and interpretation and writing-review and editing. All authors have read and approved the final version of the manuscript.
